# Directional Movement of Droplets in Grooves: Suspended or Immersed?

**DOI:** 10.1038/srep18836

**Published:** 2016-01-08

**Authors:** Wei Xu, Zhong Lan, Benli Peng, Rongfu Wen, Yansong Chen, Xuehu Ma

**Affiliations:** 1 State Key Laboratory of Fine Chemicals, Liaoning Provincial Key Laboratory of Clean Utilization of Chemical Resources, Institute of Chemical Engineering, Dalian University of Technology, Dalian 116024, China

## Abstract

The behavior of droplets trapped in geometric structures is essential to droplet manipulation applications such as for droplet transport. Here we show that directional droplet movement can be realized by a V-shaped groove with the movement direction controlled by adjusting the surface wettability of the groove inner wall and the cross sectional angle of the groove. Experiments and analyses show that a droplet in a superhydrophobic groove translates from the immersed state to the suspended state as the cross sectional angle of the groove decreases and the suspended droplet departs from the groove bottom as the droplet volume increases. We also demonstrate that this simple grooved structure can be used to separate a water-oil mixture and generate droplets with the desired sizes. The structural effect actuated droplet movements provide a controllable droplet transport method which can be used in a wide range of droplet manipulation applications.

The dynamic behavior of droplets has drawn much attention in recent years due to its fundamental effect in many practical applications. Generally, droplets can be manipulated by a surface free energy gradient^1^,^2^,^3^,^4^,^5^,^6^,^7^,^8^, temperature gradient^9^,^10^, or forces in confined geometric structures^11^,^12^,^13^. Prior to our better understanding of the capillary effect between droplets and geometric structures, shorebirds have been using their long beaks to drink water^14^,^15^,^16^,^17^ for centuries and the Cotula fallax plant in South Africa collects and retains water droplets on the foliage by a unique three-dimensional hierarchical structure formed by its leaves and fine hairs^18^. These natural phenomena have led to various structured substrates and micro systems to manipulate droplets^19^,^20^,^21^,^22^,^23^,^24^,^25^,^26^,^27^. For the shorebirds^14^,^15^,^16^,^17^, the Cotula fallax plant^18^ and a fog-collecting device^27^, the most important objective is to collect droplets from the outside of the structure. In contrast, a reversed transport direction is required for some practical applications, such as the removal of condensate droplets from micro-finned tubes, the formation of Cassie droplets on nano-array surfaces, and directional droplet transport in microfludics. Many systems can use controllable directional droplet transport via simple geometric structures with adjustable structural parameters where the transport direction is also changeable.

This study shows that the directional movement of droplets can be realized in a V-shaped groove and the movement direction can be controlled by adjusting the surface wettability of the groove inner wall and the cross sectional angle of the V-shaped groove. In contrast to the droplet transport direction in natural structures^14^,^15^,^16^,^17^, droplets in superhydrophobic grooves translate from the immersed state to the suspended state as the cross sectional angle of the V-shaped groove decreases and suspended droplets will depart from the groove bottom as the droplet volume increases. The droplet resting state and dynamic behavior presented here provide insight into the classic Cassie^28^ and Wenzel^29^ wetting modes for droplets on rough surfaces^30^,^31^,^32^,^33^,^34^,^35^,^36^,^37^,^38^. The reversed droplet transport direction, which may be a nightmare for shorebirds, actually provides an alternative method for droplet manipulation which can be used in applications such as water-oil separation and for generating droplets with desired sizes.

## Results

### Experimental verification of the angle criterion for the droplet resting states

**Figure 1:**
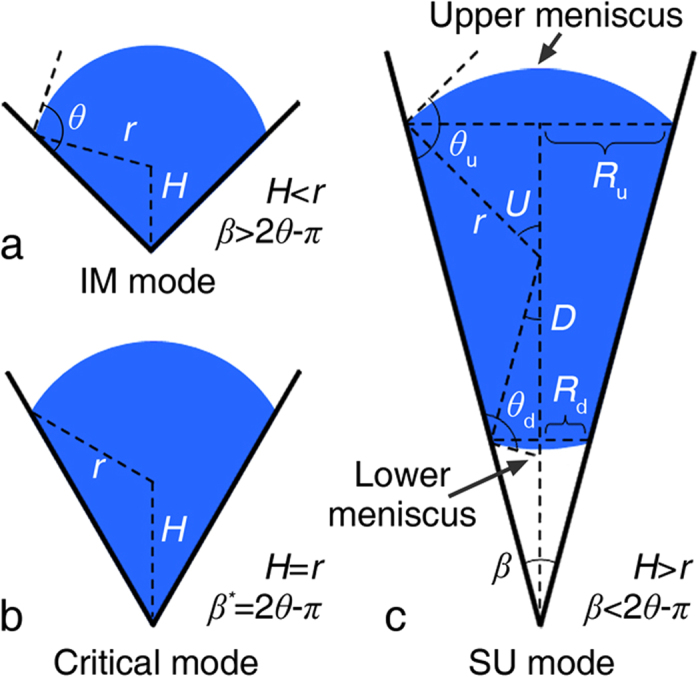
Schematic diagram of droplets in different resting modes. (**a**) The droplet immersed in the groove bottom when *β* > 2*θ*-*π*. (**b**) Droplet in between the immersed and suspended modes, as indicated by the fact that *H* = *r*. (**c**), Droplet suspended in the groove center when *β* < 2*θ*-*π*. Two meniscuses formed for case c, where *θ*_u_ and *θ*_d_ stand for the local contact angle of the upper and lower meniscuses, and *θ*_u_ = *θ*_d_ = *θ* when the droplet is in the stable SU state.

The analysis starts from the basic resting states of droplets on various grooved structures with different cross sectional angles and surface wettabilities. Figure 1 shows the possible resting states of droplets in V-shaped grooves, where the grooved structure is characterized by the apparent contact angle of the groove inner wall, *θ*, and the cross sectional angle, *β*. The IM mode denotes a droplet that is immersed in the groove bottom while the SU mode denotes a droplet that is suspended in the groove center. Due to the interfacial free energy minimization, the droplet will remain in spherical shape with identical contact angles for the local contact lines. As a result, for the suspended droplet as demonstrated in Fig. 1c, the local contact angles of the upper and lower meniscus, *θ*_u_ and *θ*_d_, will be equal to each other with *θ*_u_ = *θ*_d_ = *θ*. For the SU droplet, the half central angle of the lower meniscus, *D*, can be expressed as follows from geometry:


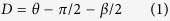


To form a suspended droplet, a lower meniscus has to form so that the groove bottom is not entirely wetted by the droplet, suggesting that the half central angle of the lower meniscus has to be larger than zero. The following criterion can then be obtained:


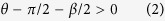


Equation (2) predicts that a droplet will be suspended in the groove center for a groove with *β* < 2*θ*-*π*, as shown in Fig. 1c.

**Figure 2:**
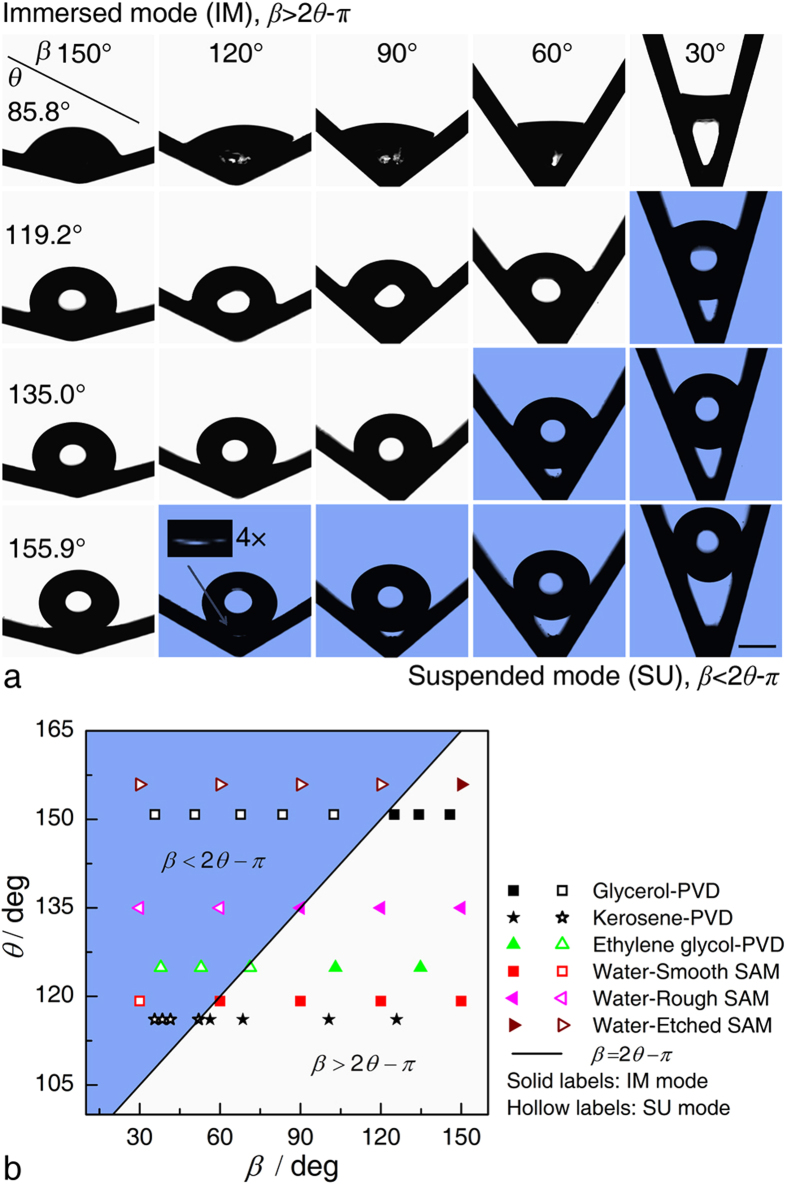
Relationship between the groove parameters and the droplet resting modes. (**a**) Experimental images of water droplets in the V-shaped grooves with various surface wettabilities and cross sectional angles. (**b**) Relationship between the groove parameters and the droplet resting modes for water, glycerol, kerosene, and ethylene glycol droplets. The droplets will be either immersed or suspended in the grooves depending on the combination of *θ* and *β*. For *β* < 2*θ*-*π*, the droplets were suspended in the groove center for all the liquids under consideration. The scale bar denotes 1 mm.

The criterion in Eq. (2) was then tested by a series of experiments with four grooved surfaces, Smooth, Smooth SAM (Self-Assembled Monolayer), Rough SAM, and Etched SAM surfaces (Methods). The surface wettabilities, including the apparent contact angle, *θ*, advancing contact angle, *θ*_a_, and receding contact angle, *θ*_r_, are listed in Table 1. Experimental images of the droplets in different grooves are shown in Fig. 2a. As expected, two resting states are observed depending on the combination of the contact angle, *θ*, and the cross sectional angle, *β*. For smaller *θ* and larger *β* conditions, the droplets are immersed within the grooves in the Immersed (IM) mode. For other conditions with *β* < 2*θ*-*π*, the droplets are suspended in the groove center in the Suspended (SU) mode. These experimental results provide support for the criterion in Eq. (2) and the predictions for the angular liquid bridge made by Concus and Finn^39^. In addition to the resting states of water droplets, the resting states of glycerol, kerosene and ethylene glycol droplets with relatively lower surface free energies were also observed on the PVD (Physical Vapor Deposition, Methods) treated grooves. The results in Fig. 2b indicate that the analysis also applies to other liquids.

### Effect of β on the resting mode transitions and directional movements

**Figure 3:**
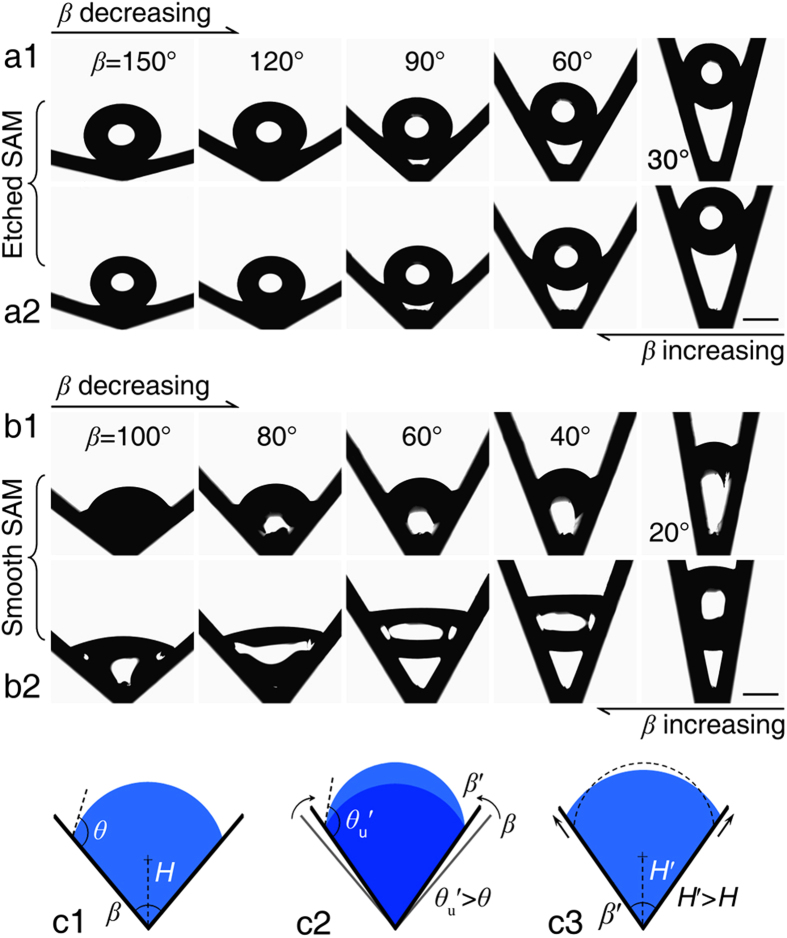
IM-to-SU and SU-to-IM resting mode transitions for the Etched SAM and Smooth SAM grooves as *β* changed. (**a1)** The IM-to-SU transition occurs around *β* = 106° for the Etched SAM groove as *β* decreases from high-to-low. (**a2)** The SU-to-IM transition occurs around *β* = 109° as *β* increases. (**b1)** The IM-to-SU transition is not observed in the Smooth SAM groove when *β* is decreasing from high-to-low. (**b2)** The SU-to-IM transition occurs around *β* ~ 70° when *β* is increasing. The difference between b1 and b2 indicates that the droplet resting state is also affected by the contact angle hysteresis. (**c1**) Schematic diagram of a droplet in the stable IM mode. (**c2**) When *β* decreases slightly to *β*′, the upper meniscus is deformed and the local contact angle increases from *θ* to *θ*_u_′. (**c3**) *θ*_u_′ reaches *θ*_a_, which drives the upper meniscus contact line to move upward. The driving force for the IM-to-SU transition is provided by the meniscus deformation. The scale bar denotes 1 mm.

The resting mode transitions and directional droplet movements can be realized by dynamically adjusting the cross sectional angle of the grooves. The Etched SAM and Smooth SAM grooves were used in these experiments with *β* changing from high-to-low and low-to-high (Methods). The resultant experimental images are shown in Fig. 3. For the Etched SAM groove, the IM-to-SU transition occurred around *β* = 106° (Fig. 3a1, Supplementary Movie 1), while the SU-to-IM transition occurred around *β* = 109° (Fig. 3a2, Supplementary Movie 2). The resting mode and the droplet profiles for the same *β* are similar when *β* is adjusted in opposite directions. The results in Fig. 3b2 also show that the droplet departed from the groove bottom with decreasing *β*. This phenomenon is in contrast with that happens in shorebirds when they use their long beaks to drink water. In their case, the beak acts as a V-shaped groove and the droplet is transported from the tip of the beak into the mouth as the shorebird closes its beak. Our analyses demonstrate that the droplet moving direction changes when the surface wettability of the groove inner wall is modified from hydrophilic (Shorebird beaks) to superhydrophobic (Etched SAM groove).

The resting mode transition and droplet movement are also strongly affected by the contact angle hysteresis. For the Smooth SAM groove with the relatively higher contact angle hysteresis, the SU mode is expected when *β* becomes smaller than 58.4° according to Eq. (2), but the IM-to-SU transition was not observed with decreasing *β* (Fig. 3b1, Supplementary Movie 3). However, the SU-to-IM transition occurred around *β* ~ 70° when *β* was increased from low-to-high (Fig. 3b2, Supplementary Movie 4). The results indicate that the droplet resting mode is not only controlled by *θ* and *β*, but also by the contact angle hysteresis. The latter effect can be directly observed in Fig. 3 for the Smooth SAM groove. When *β* is increasing from low-to-high, the droplet is stretched into an irregular shape (Fig. 3b2) and the local contact angle of the upper meniscus, *θ*_u_, is less than 90°, indicating that the contact line of the upper meniscus is restricted by *θ*_r_ when the suspended droplet tries to wet the groove bottom. The contact angle hysteresis also explains why the IM-to-SU transition was not observed for the Smooth SAM groove when *β* was decreased from high-to-low.

The underlying mechanism for the resting mode transition is schematically demonstrated in Fig. 3. Consider a droplet in the IM mode (Fig. 3c1), when *β* decreases slightly to *β*′, the contact line will not move immediately due to the contact angle hysteresis. Instead, the upper meniscus will be deformed and the local contact angle will increase from *θ* to *θ*_u_′ (Fig. 3c2). As *β* decreases even further, *θ*_u_′ increases to *θ*_a_, which drives the contact line of the upper meniscus to move forward. Although the resultant droplet in Fig. 3c3 is still in the IM mode, the center of curvature of the immersed droplet is moving away from the groove bottom, as indicated by the fact that *H*′ > *H*. Here, *H* is defined as the distance between the curvature center of the droplet and the groove bottom. The movement of the upper meniscus continues as *β* further decreases and *H* increases. The continuous movement of the upper meniscus is finally able to drag the droplet away from the groove bottom to form a suspended droplet. The IM-to-SU transition occurs because the droplet in SU mode is energetically more favorable when *β* is decreased.

This analysis indicates that the driving force for the resting mode transition is provided by the deformation of the upper meniscus. However, the formation of the lower meniscus is restricted by the contact angle hysteresis, as shown in Fig. 3b1. For a droplet undergoing the IM-to-SU transition, the basic requirement for lower meniscus formation is that the liquid adjacent to the groove bottom has a contact angle that becomes larger than the receding angle, *θ*_r_. Thus, the critical condition for the IM-to-SU transition is:


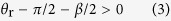


Equation (3) indicates that the receding angle, *θ*_r_, has to be larger than 90° to realize the IM-to-SU transition. As a result, the IM-to-SU transition will not occur on the Smooth SAM groove with *θ*_r_ = 67.9°, but will occur on the Etched SAM groove with *θ*_r_ = 152°. The analyses indicate that the immersed droplet can be transformed to a suspended droplet and the inside-out droplet movement can be realized by dynamically reducing the cross sectional angle of the V-shaped groove. To ensure this directional movement, a relatively higher contact angle and less contact angle hysteresis are required on the groove inner wall.

### Spontaneous movements as the droplet volume changes

The directional droplet movement can also occur spontaneously when the grooved structure remains fixed and no external forces are applied. For practical applications, the grooves are usually fixed while the droplet volume is increased or decreased by vapor condensation or evaporation. The effects of the capillary force and the geometric structure of the V-shaped groove can cause spontaneous motion of the droplet as the droplet volume changes.

**Figure 4:**
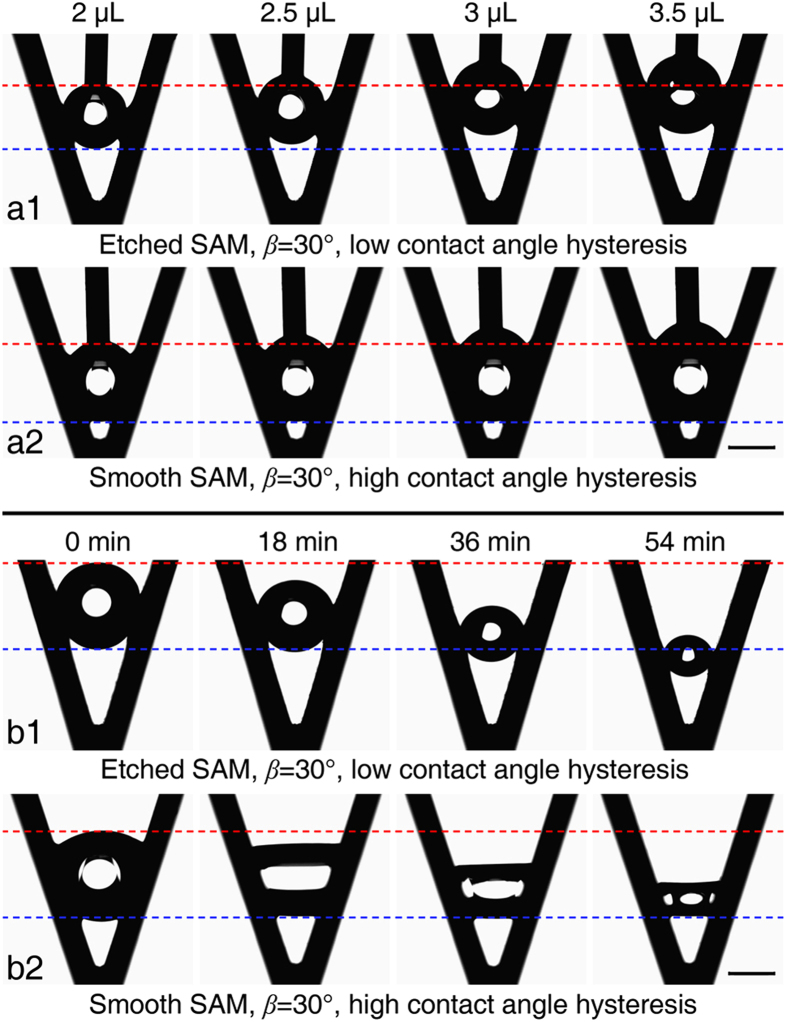
Dynamic droplet movements within the Etched SAM and Smooth SAM grooves during simulated growth and evaporation. (**a1**) The upper and lower meniscuses move upward simultaneously as the droplet volume increases, causing the droplet to depart from the Etched SAM groove bottom. (**a2**) For the Smooth SAM groove, the upper meniscus moves upward as the droplet volume increases, while the lower meniscus stays fixed. (**b1**) The droplet in the Etched SAM groove moves downward during evaporation with the upper and lower meniscuses retracting almost simultaneously. (**b2**) For the Smooth SAM groove, the upper meniscus retracts into the groove bottom during evaporation, while the lower meniscus stays fixed. The scale bar denotes 1 mm.

Figure 4 shows the growth and decay of droplets in the Etched SAM and Smooth SAM grooves with *β* = 30° (Methods). For the Etched SAM groove, the differences between *θ*_a_, *θ*_r_ and *θ* are all small enough to ensure that the upper and lower meniscuses move upward almost simultaneously. As the droplet volume increases, the upper and lower meniscuses all move upward so that the droplet departs from the groove bottom and the resultant larger droplet is suspended higher in the groove (Fig. 4a1, Supplementary Movie 5).

The spontaneous droplet movement is also strongly affected by the contact angle hysteresis. For the Smooth SAM groove, the droplet growth results in movement of the upper meniscus, while the lower meniscus contact line does not move, and the resulting larger droplet is in the stretched SU mode (Fig. 4a2, Supplementary Movie 6). The upper and lower contact lines will move when the contact angles reach *θ*_a_ and *θ*_r_. For the Smooth SAM groove, *θ*_a_ is easily achieved because the difference between *θ*_a_ and *θ* is relatively small, while *θ*_r_ cannot be reached during the droplet growth. As a result, the upper contact line keeps moving upward while the lower one remains fixed. Comparison of the droplet movement in the Etched SAM and the Smooth SAM grooves indicates that a relatively higher contact angle and less contact angle hysteresis are also required for the groove inner wall to promote effective spontaneous droplet movement.

Similar phenomena were observed during evaporation (Methods). For the Etched SAM groove, the upper and lower contact lines move downwards almost simultaneously as during the droplet growth due to the relatively small contact angle hysteresis (Fig. 4b1). For the Smooth SAM groove, the upper and lower meniscuses simultaneously deform, with the receding contact angle of the upper meniscus, *θ*_r_, reached first, which pushes the upper contact line to move downward, while the lower meniscus contact line remains fixed (Fig. 4b2).

The underlying mechanism for this phenomenon can also be explained by the schematic in Fig. 3, except for the presence of the lower meniscus and the fact that the meniscus deformation is caused by the droplet volume increase instead of the variation of *β*. For a SU mode droplet, the droplet volume increase will first cause deformation of the meniscuses and the local contact angle will then increase. The local contact angles for the newly-deformed upper and lower meniscuses, *θ*_u_′ and *θ*_d_′, are related as (see the Supplementary Figure 1 and Supplementary Discussion 1):


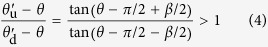


The result *θ*_u_′ > *θ*_d_′ indicates that the contact angle of the upper meniscus is increasing more rapidly and is approaching *θ*_a_ as the droplet volume increases. As a result, the contact line for the upper meniscus starts to move upward as the droplet volume increases, as shown in Fig. 4a2. A similar analysis can be easily applied to the evaporation process, with the contact angle of the upper meniscus first approaching the receding angle, which pushes the contact line of the upper meniscus to move downward (Fig. 4b2).

**Figure 5:**
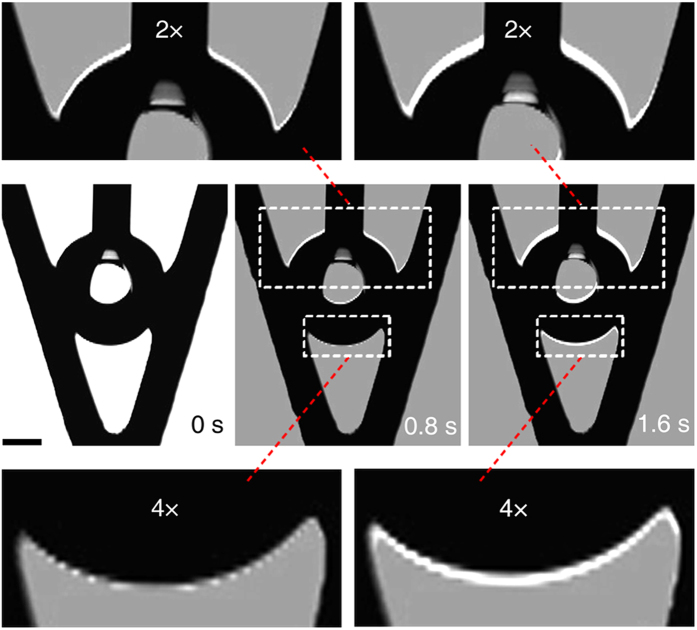
Overlaid images of the sequential meniscus movement. The images captured at *t* = 0.8 s and 1.6 s are overlaid onto the *t* = 0 s image for comparison. The white area indicates the local contact line movement. The upper and lower meniscuses are magnified by 2 and 4 times for clarity. At 0.8 s, the upper meniscus advances due to the increased droplet volume, while the lower meniscus stays fixed. At 1.6 s, the upper meniscus continues to move upward and the lower meniscus starts to move upward, driving the droplet away from the groove bottom. The upper and lower meniscuses move in unison. The scale bar denotes 0.5 mm.

The results agree well with detailed droplet growth observations for the Etched SAM groove, as demonstrated in Fig. 5. The droplet movement is in stepwise manner and Fig. 5 shows one cycle of this stepwise movement. Considering that the droplet moving distance in one cycle is small, the upper and lower meniscus images that captured at different times are overlaid layer by layer (Methods) to show the movements. The contact line moves as predicted. At *t* = 0.8 s, the upper meniscus first moves upward as indicated by the white area on the overlaid image, while the lower meniscus stays fixed. 0.8 s later, the upper meniscus keeps moving upward and the lower meniscus has also started to move upward. This result suggests that the driving force for the lower meniscus movement is provided by the dynamic movement of the upper meniscus. As the contact line moves upward, the upper and lower meniscuses will deform again in relation to where the newly formed contact line is located. The resultant local contact angle of the lower meniscus may reach *θ*_r_ for grooves with low contact angle hysteresis and the lower meniscus will move upward. As a result, the droplet is suspended even higher.

The analyses demonstrate that, for grooves with relatively high contact angles and small contact angle hysteresis, the suspended droplet will keep climbing up from the groove bottom during the droplet growth. This phenomenon is spontaneous and provides a passive method for droplet manipulation applications such as the removal of condensate droplets from micro-finned tubes.

### Potential applications

**Figure 6:**
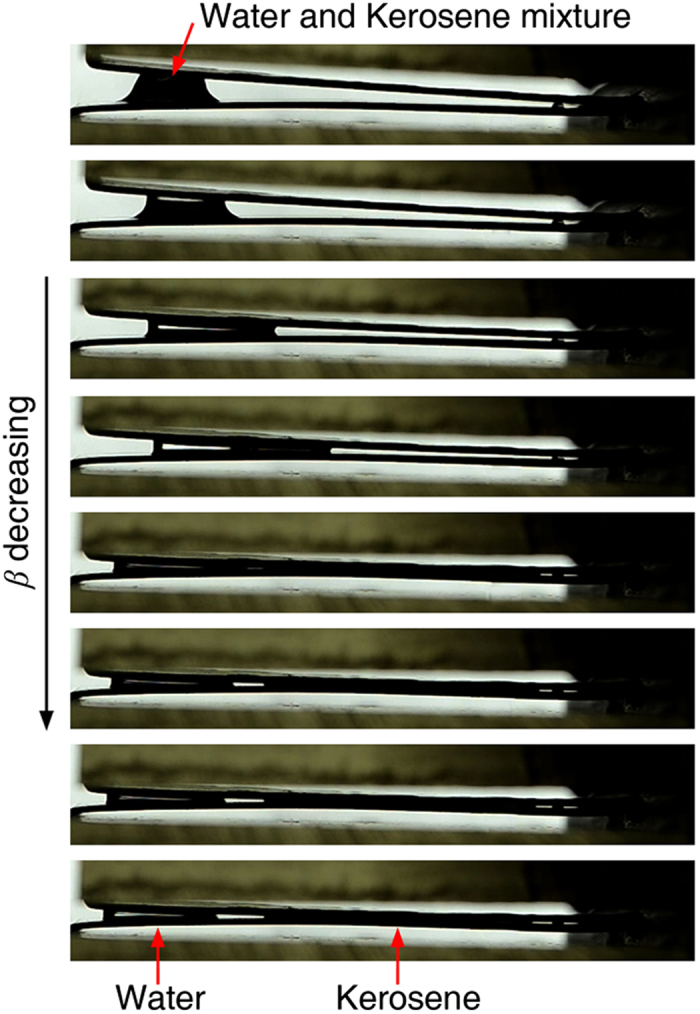
Water-kerosene separation by a V-shaped structure. The V-shaped structure can be used to separate water and kerosene mixture. As the cross sectional angle of the V-shaped structure decreases, the water and kerosene move in opposite directions and finally separate into two parts.

**Figure 7:**
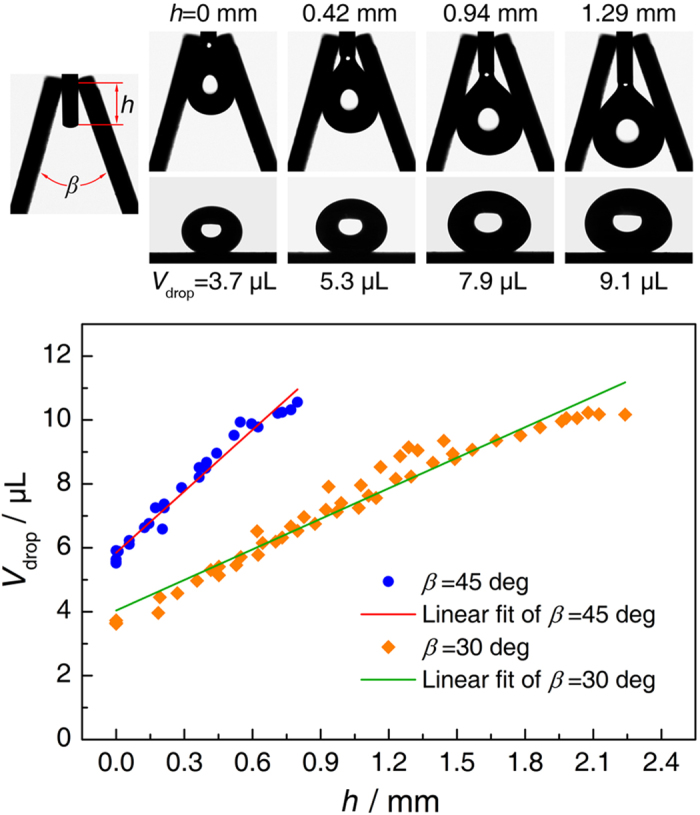
Generating droplets with desired sizes by a V-shaped structure. A V-shaped structure is mounted on a stainless steel microsyringe needle and the volume of the departing droplet can be easily adjusted. The volume of the departing droplet is a function of the cross sectional angle, *β*, and the distance between the needle tip and the groove bottom, *h*.

The study provides a droplet manipulation method which can be used in a wide range of applications such as wetting mode regulation, water-oil separation (Fig. 6), generating droplets with desired sizes (Fig. 7), rapid removal of condensate droplets during dropwise condensation and other applications in microfludics. In Fig. 6, tests have shown that the V-shaped structure can be used to separate a water-kerosene mixture. For water, the criterion in Eq. (2) is fulfilled and a water droplet is formed as the cross sectional angle of the V-shaped groove decreases. A *β* decrease even further, the water and kerosene move in opposite directions and finally separate into two parts.

The V-shaped structure can also be used to generate droplets with different sizes. We have adopted the V-shaped structure to generate droplets in measuring the contact angle of superhydrophobic surfaces. During the contact angle measurement, the droplet generated by the stainless steel microsyringe needle is usually ~10 μL, while a droplet with a volume of 3 ~ 5 μL is usually desired to minimize the gravitational effect. Figure 7 shows that the volume of a departing droplet from the microsyringe needle can be easily adjusted by using V-shaped grooves with different geometric conditions. The experimental results show that the volume of the departing droplet is a function of the cross sectional angle, *β*, and the distance between the needle tip and the groove bottom, *h*. The droplet volume increases lineally with *h* and decreases when *β* is decreased, providing an effective method to generate droplets with desired sizes. The structure also can be used as a dosing device to generate droplets with desired sizes in microfluidics and microreactors. Moreover, the V-shaped structure also can be used to realize the directional movement of condensate droplets during dropwise condensation, which is able to accelerate the departure of condensate droplets and improve the heat transfer performance.

## Discussion

For the droplets in V-shaped grooves, the resting states and the dynamic behaviors of droplets are mainly affected by two factors, including the surface wettabilities (contact angle and contact angle hysteresis) and the geometric parameters. A better understanding of the capillary effect between droplets and geometric structures is certainly helpful for the droplet manipulation applications.

The present work shows that the resting states of droplets in grooves can be predicted by a criterion of *β* < 2*θ*-*π*, with the suspending droplets formed in grooves with superhydrophobic inner walls and small cross sectional angles. The resting state also changes with the geometric parameters. For instance, the droplet in a superhydrophobic groove can translate from the immersed state to the suspended state as the cross sectional angle of the groove decreases. The IM-to-SU transition occurs because the droplet in SU mode will be energetically more favorable when *β* is decreased. The driving force for the IM-to-SU transition is provided by the deformation of the upper meniscus when the groove cross sectional angle is decreasing.

In addition, the directional movement of droplets can be realized in the V-shaped groove and the moving direction is also changeable. Our analyses demonstrate that the movement of droplet meniscuses are in stepwise manner and can be affected greatly by the contact angle hysteresis. In a superhydrophobic groove, the suspended droplet departs from the groove bottom as the droplet volume increases. This structural effect actuated droplet movements offer new insights into the interfacial phenomena of droplets trapped in geometric structures and provide a controllable droplet transport method which can be used in a wide range of droplet manipulation applications.

## Methods

### Experimental system

The experimental setup was composed of a grooved structure, a microsyringe system, a mechanical stepping drive, and a microscopic CCD camera (Supplementary Figure 2). The grooved structure was folded from a copper sheet and connected to stepping drives on each side. In this way, the cross section angle *β* of the groove could be adjusted in real time to the desired values. The microsyringe system was used to generate droplets with the desired volumes for the wetting mode measurements and to adjust the droplet volume in real time for the droplet growth observations. The surface wettabilities, including the apparent contact angle *θ*, advancing contact angle *θ*_a_, and receding contact angle *θ*_r_, were measured by an OCAH200 contact angle system (Dataphysics, Germany, ±0.1°) with the results listed in Table 1. The experiments are conducted in an ambient environment with room temperatures of 18~24 °C and relative humidities of 35~45% RH.

### Experimental procedure

The droplet growth was simulated with a 2 μL water droplet placed in the groove with a microsyringe needle then inserted into the droplet to supply water at a constant flow rate of 0.06 μL/s. The effect of the inserted needle on the droplet movement can be neglected since its contact angle is ~90° and it has a relatively small diameter. In the evaporation experiments, a 4 μL water droplet was placed in the groove and then the droplet was allowed to evaporate by exposure to the ambient environment.

### Imaging

The experimental images were captured by the CCD camera of the OCAH200 system with a resolution of 752 × 484 pixels and frame rate of 60 fps. The image overlapping in Fig. 5 was conducted in Photoshop with the “Difference” blend mode.

### Materials

The V-shaped grooved structures were folded from a copper sheet. The groove surfaces (inner walls) were treated with W0.5 abrasive paste polishing, 180# sandpaper grinding, and chemical etching^40^ followed by the addition of a self-assembled monolayer (SAM) of n-Octadecyl mercaptan^41^. The fabricated groove structures are referred to as Smooth, Smooth SAM, Rough SAM, and Etched SAM grooves depending on the fabrication method. The PVD surface was fabricated using physical vapor deposition^42^ of 1H, 1H, 2H, 2H-Perfluorooctyltrichlorosilane on the chemically etched copper surface^40^. Deionized water, glycerol, kerosene and ethylene glycol were used to generate the droplets and for observations of the resting state.

## Additional Information

**How to cite this article**: Xu, W. *et al*. Directional Movement of Droplets in Grooves: Suspended or Immersed? *Sci. Rep.* **6**, 18836; doi: 10.1038/srep18836 (2016).

## Supplementary Material

Supplementary Information

Supplementary Movie S1

Supplementary Movie S2

Supplementary Movie S3

Supplementary Movie S4

Supplementary Movie S5

Supplementary Movie S6

## Figures and Tables

**Table 1:** Contact angles on the groove inner walls.

Grooves	*θ* (deg)	*θ*_a_ (deg)	*θ*_r_ (deg)
Smooth	85.8	96.2	~30
Smooth SAM	119.2	124.3	67.9
Rough SAM	135.0	138.1	~45
Etched SAM	155.9	~158	~152
