# Supplementary material for: Directional Movement of Droplets in Grooves: Suspended or Immersed?
Source: Sci Rep. 2016 Jan 8;6:18836. doi: 10.1038/srep18836 (PMC4705533; doi:10.1038/srep18836)
Supplement: Supplementary Information [file srep18836-s1.pdf]

Supplementary Information

Directional Movement of Droplets in Grooves: Suspended or Immersed?

Wei Xu[†], Zhong Lan[†], Benli Peng, Rongfu Wen, Yansong Chen and Xuehu Ma*

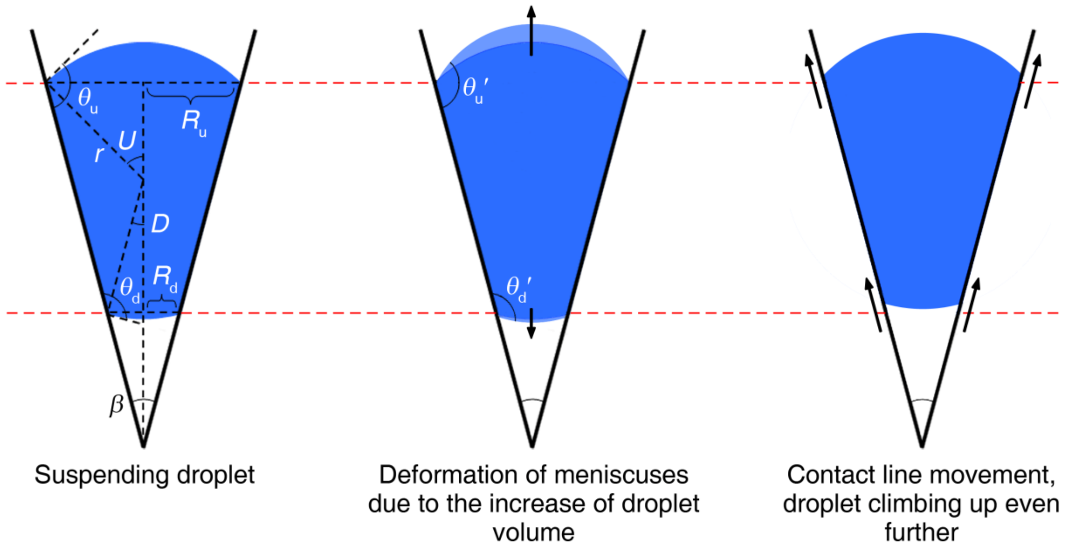

Supplementary Figure 1 Schematic diagram of the droplet movement mechanism as the droplet volume increases.

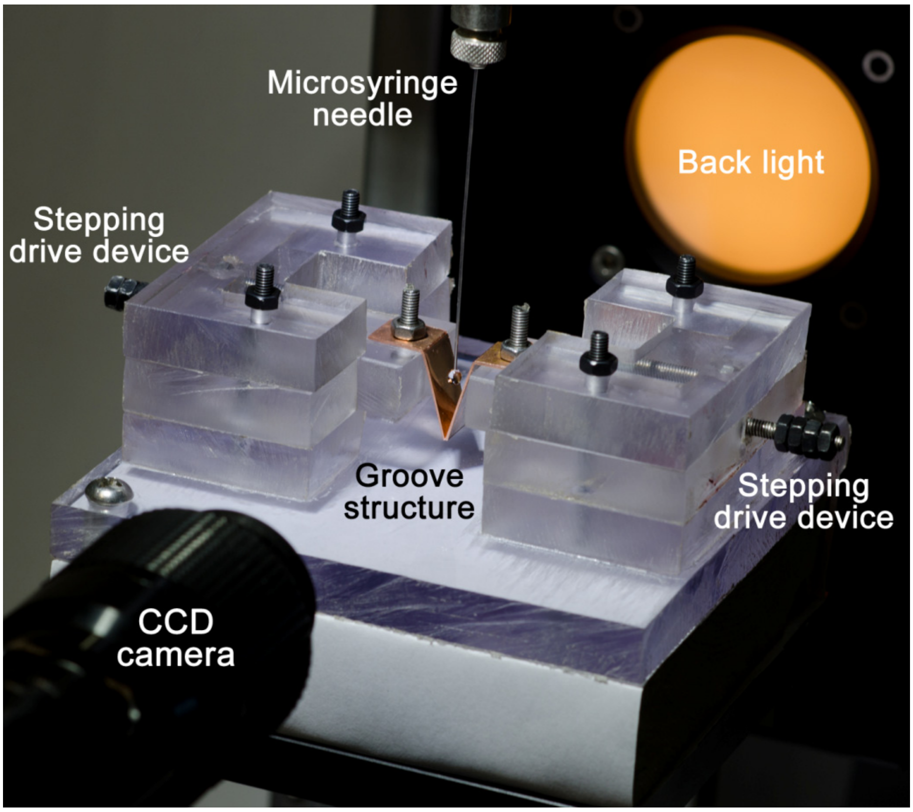

Supplementary Figure 2 Experimental apparatus.

Supplementary Discussion 1 Model analysis of the droplet movement mechanism as the droplet volume increases.

Consider a droplet in suspended mode as demonstrated in Supplementary Fig. 1, and neglect the effect of gravitational force for simplicity. The following relationship can be obtained:

$$\frac{R_u}{R_d}=\frac{\sin(\theta-\pi/2+\beta/2)}{\sin(\theta-\pi/2-\beta/2)} \tag{1}$$

where R_u and R_d are the bottom radii of the upper and lower meniscuses.

The droplet volume increase will cause first deformation of the meniscuses. Defining that the local contact angles of the newly-deformed upper and lower meniscuses as θ_u' and θ_d' (Supplementary Fig. 1). Then equation (1) for the newly-deformed meniscuses can be written as:

$$\frac{R_u}{R_d}=\frac{\sin(\theta_u'-\pi/2+\beta/2)}{\sin(\theta_d'-\pi/2-\beta/2)} \tag{2}$$

Associating equations (1) and (2) and applying the Euler method for a relatively small change between θ' and θ give:

$$\frac{R_u}{R_d}=\frac{\cos(\theta-\pi/2+\beta/2)\cdot(\theta_u'-\theta)}{\cos(\theta-\pi/2-\beta/2)\cdot(\theta_d'-\theta)} \tag{3}$$

Substituting equation (1) into equation (3) give:

$$\frac{\theta_u'-\theta}{\theta_d'-\theta}=\frac{\tan(\theta-\pi/2+\beta/2)}{\tan(\theta-\pi/2-\beta/2)}>1 \tag{4}$$

The result of $\theta_u'>\theta_d'$ indicates that the contact angle of the upper meniscus is increasing more rapidly and approaching θ_a as the droplet volume increases. As a result, the contact line on the upper meniscus starts to move forward first as the droplet volume increases.

Supplementary Movie 1 IM-to-SU resting mode transition occurs around 106 degree for the Etched SAM groove when the cross sectional angle decreases.

Supplementary Movie 2 SU-to-IM resting mode transition occurs around 109 degree for the Etched SAM groove when the cross sectional angle increases.

Supplementary Movie 3 IM-to-SU resting mode transition fails for the Smooth SAM groove when the cross sectional angle decreases from high-to-low.

Supplementary Movie 4 SU-to-IM resting mode transition occurs around 70 degree for the Smooth SAM groove when the cross sectional angle increases.

Supplementary Movie 5 Simulated droplet growth on the Etched SAM groove.

Supplementary Movie 6 Simulated droplet growth on the Smooth SAM groove.
