# Supplementary figures and images for: Directional Movement of Droplets in Grooves: Suspended or Immersed?
Source: Sci Rep. 2016 Jan 8;6:18836. doi: 10.1038/srep18836 (PMC4705533; doi:10.1038/srep18836)

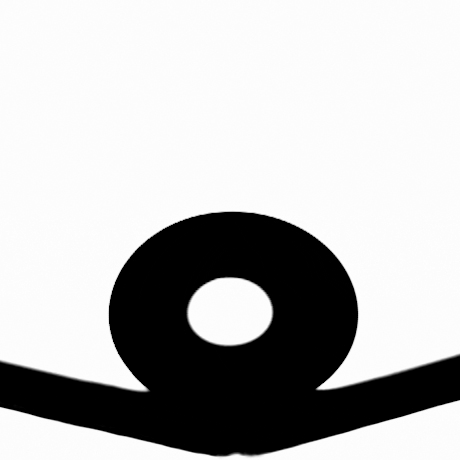

Supplement: Supplementary Movie S1 [file srep18836-s2.gif]

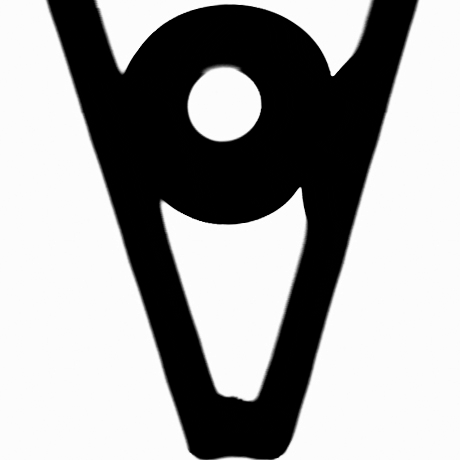

Supplement: Supplementary Movie S2 [file srep18836-s3.gif]

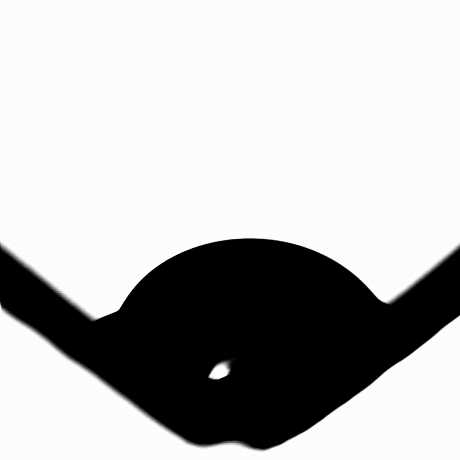

Supplement: Supplementary Movie S3 [file srep18836-s4.gif]

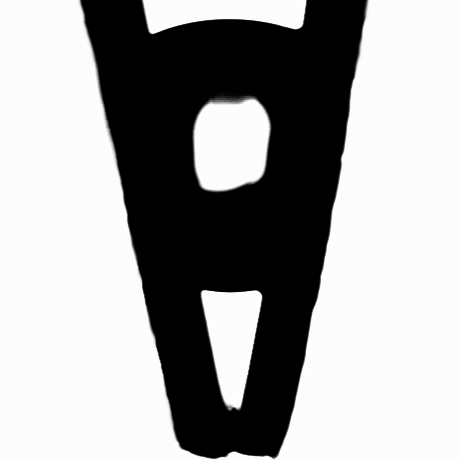

Supplement: Supplementary Movie S4 [file srep18836-s5.gif]

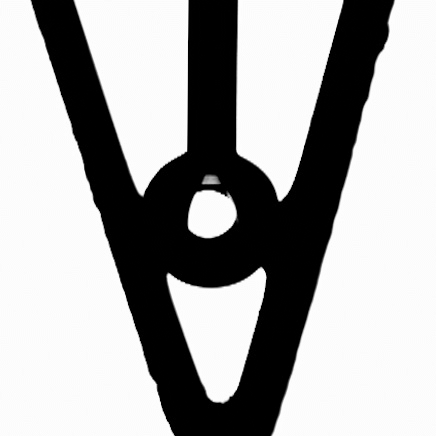

Supplement: Supplementary Movie S5 [file srep18836-s6.gif]

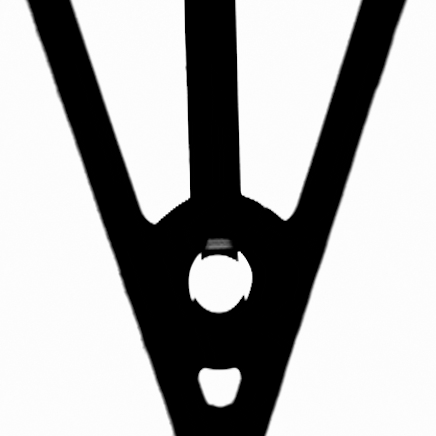

Supplement: Supplementary Movie S6 [file srep18836-s7.gif]
